# Novel Image Processing Method for Detecting Strep Throat (Streptococcal Pharyngitis) Using Smartphone

**DOI:** 10.3390/s19153307

**Published:** 2019-07-27

**Authors:** Behnam Askarian, Seung-Chul Yoo, Jo Woon Chong

**Affiliations:** 1 Department of Electrical and Computer Engineering, Texas Tech University, Lubbock, TX 79409, USA; 2 School of Communication & Media, Ewha Womans University, Seoul 03760, Korea

**Keywords:** strep throat, image processing, color space, classification, smartphone

## Abstract

In this paper, we propose a novel strep throat detection method using a smartphone with an add-on gadget. Our smartphone-based strep throat detection method is based on the use of camera and flashlight embedded in a smartphone. The proposed algorithm acquires throat image using a smartphone with a gadget, processes the acquired images using color transformation and color correction algorithms, and finally classifies streptococcal pharyngitis (or strep) throat from healthy throat using machine learning techniques. Our developed gadget was designed to minimize the reflection of light entering the camera sensor. The scope of this paper is confined to binary classification between strep and healthy throats. Specifically, we adopted *k*-fold validation technique for classification, which finds the best decision boundary from training and validation sets and applies the acquired best decision boundary to the test sets. Experimental results show that our proposed detection method detects strep throats with 93.75% accuracy, 88% specificity, and 87.5% sensitivity on average.

## 1. Introduction

According to the U.S. National Health Statistics Report, strep throat (*streptococcal pharyngitis*) is one of the main reasons for patient visits to hospital emergency departments in the U.S. [1]. Strep throat is an infection that is caused by bacteria [2]. Specifically, Group A beta-hemolytic streptococcus is the main cause of streptococcal pharyngitis in children and adults [3,4]. One of the risks of late strep throat diagnosis is rheumatic fever, which may lead to chronic rheumatic heart disease [5]. Rheumatic fever is the cause of death for approximately 320,000 patients a year globally [6,7]. Hence, early diagnosis of strep throat is crucial for preventing deaths related to rheumatic heart disease, especially in remote areas with a medical shortage. Moreover, a false diagnosis of strep throat may cause inappropriate treatment using antibiotics that would lead to bacterial resistance [8,9].

The common diagnosis method is the clinical decision utilizing the Centor score that is calculated from a set of criteria which includes coughing, fever, etc. [2,3,5,7,8,10]. However, its accuracy is less than 86% [10,11]. Throat culture is another clinical diagnosis method detecting streptococcal pharyngitis [9,11,12,13,14,15,16], which adds a sample of cells from the throat to a substance to promote the growth of the bacteria and diagnoses the disease. If bacteria grows (positive), it indicates that the patient has a bacterial infection [15]. Otherwise, the patient does not have a bacterial infection. The accuracy of this culture method for strep detection is 98% [15]. Strep throat was also diagnosed with the help of touch spray ionization mass spectrometry [14]. However, these diagnosis methods need trained physicians or specialists. Hence, timely and accessible diagnosis for all patients is still a challenge.

There have been studies which use color intensity values to detect diseases like diabetes [17,18], internal-organ diseases [19,20,21], or heart and kidney diseases [17,18,22,23,24,25,26,27,28,29]. These color intensity value-based methods have been combined with machine learning techniques such as naive Bayes, Bayes net, and sequential minimal optimization (SMO) [30,31,32]. In these studies, 21 properties were extracted from tongue color intensity values to diagnose 23 different types of diseases. Despite the capability of diagnosing different diseases using tongue color features, there exist some limitations identifying syndromes, distinguishing color features, and classifying the diseases [17,22,23,24]. For example, Zhang and Kim et al., concluded that different light conditions, color spaces, and devices can make the fore-mentioned methods to be less reliable in diagnosing corresponding diseases [17,33,34]. Even though there have been studies on smartphone-based tongue color analysis for medical diagnosis [34,35] as mentioned above, to the best of the authors’ knowledge, there has been no research on smartphone-based strep throat detection using color analysis.

In this paper, we propose a novel and robust throat color analysis technique using YCbCr color space and least square estimation-based color correction method with images obtained from the smartphone camera to detect strep throat. Our proposed method uses an add-on gadget which helps to acquire throat images in an accurate manner. The YCbCr color space separates the luminance factor from the color space and makes it independent of luminance changes to detect the region of interest (ROI). The novel color correction method copes with different sensors and chroma variations to provide a unified color space. For classification, the *k*-NN classifier was adopted to distinguish healthy and diseased throat. As a result, the proposed method provides detection of strep throat with the images captured by the smartphone camera. The rest of this paper is organized as follows: Section 2 describes data collection and feature extraction. Section 3 describes the results from our proposed method, and finally Section 4 concludes the paper.

## 2. Materials and Methods

**Figure 1:**
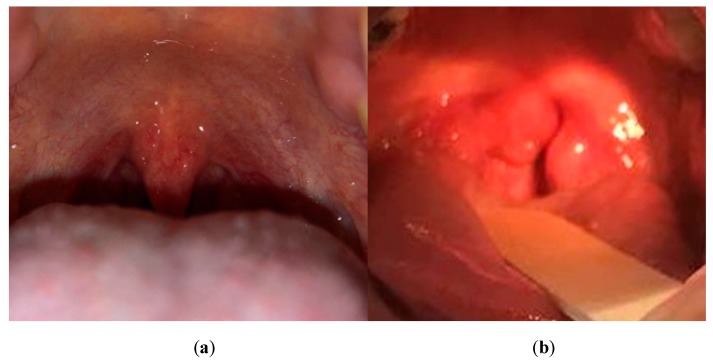
Example of a healthy and strep throat. (**a**) Healthy throat where there is no sign of any infection, and (**b**) strep throat where there are red swollen uvula and tonsils, and whitish spots on the throat.

Strep throat symptoms are inflammations, red spots on the back of the throat, and enlarged tonsils, which are shown in Figure 1b [36]. In this paper, we propose a smartphone-based strep throat detection method, which classifies strep throats from healthy throats using the image features shown in Figure 1. The classification of our proposed method is confined to binary classification between strep and healthy throats. Data acquisition required for testing the proposed method is explained in Section 2.1 while the proposed strep detection method consisting of (1) preprocessing, (2) feature extraction, and (3) classification is described in Section 2.2, Section 2.3 and Section 2.4, respectively.

### 2.1. Data Acquisition

We recruited 56 subjects following the Texas Tech University Institutional Review Board (IRB) (IRB#: IRB 2018-701). The subjects (56) consisted of 28 healthy and 28 strep throat-diagnosed subjects whose ages were in the range of 20 to 38 years old. Among 56 subjects, 31 were male and 25 were female. Subjects were asked to sit in a relaxed position without any movement and instructed to open their mouths widely. At that moment, experimenters captured subjects’ throat images using a smartphone camera. We used the iPhone X rear camera and set the resolution of the camera to its maximum resolution at 12-megapixels (4032 x 3024 pixels). We used the autofocus function of the iPhone X and turned the light emitted diode (LED) flashlight on during the image acquisition.

**Figure 2:**
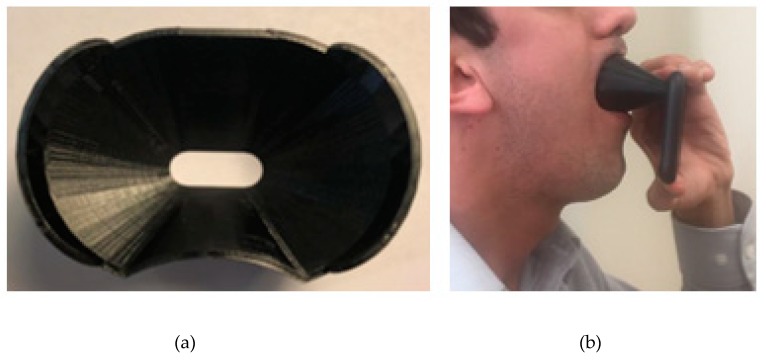
Our developed add-on gadget and its usage for data acquisition. (**a**) Add-on gadget designed and manufactured by 3D printing, and (**b**) image acquisition setup using the iPhone X with the add-on gadget.

Figure 2 shows our developed add-on gadget and its usage with the iPhone X. We designed and manufactured this add-on gadget customized to iPhone X using a 3-D printer. This gadget made the smartphone’s flashlight shine on the throat in a bright and uniform way. Moreover, it eliminated the effect of ambient light, minimized tongue movement, and prevented the tongue from blocking the throat, Figure 2. 

### 2.2. Preprocessing

The preprocessing step is needed for accurate and effective feature extraction in throat images. Two main parts of the preprocessing steps are (1) color correction and (2) image segmentation. Color correction is required to derive the output image independent from the color space since each smartphone camera has its own color space parameters [37]. On the other hand, image segmentation is required to extract a region of interest (ROI) from the input raw image since images taken by the smartphone camera may include other parts of the inner mouth (soft palate and teeth, lips, etc.). 

#### 2.2.1. Color Correction

**Figure 3:**
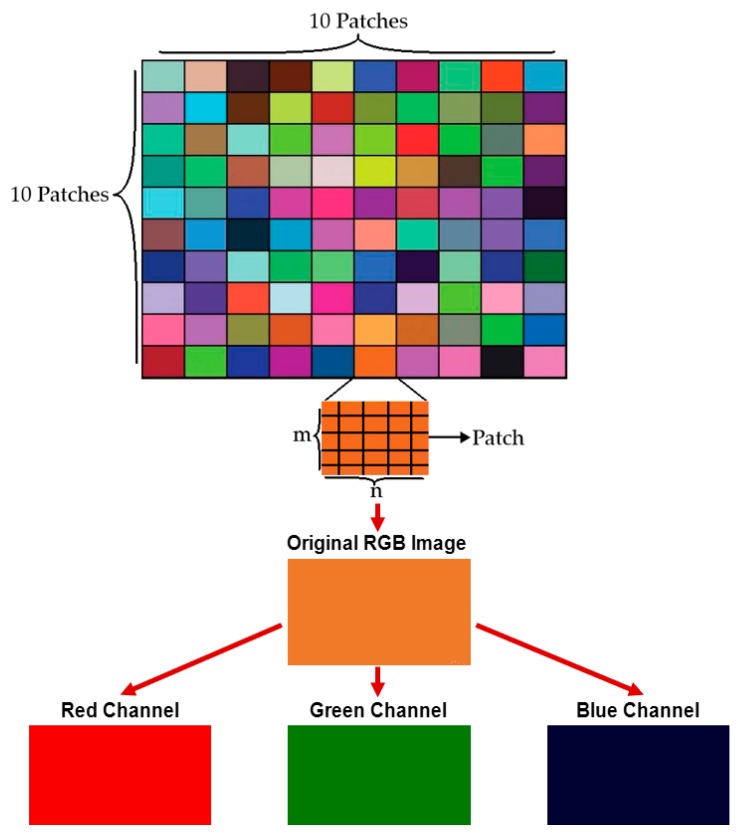
Color correction step. Color chart with 100 patches (10 × 10 patches) were generated. Each patch inside the color chart is an image with *m* × *n* pixels. Each patch is presented in its R, G, B color channel components.

For color correction, we adopted the least square estimation-based color correction method [38], which calculates color correction matrix ***A*** based on least-square estimation toward the reference color. We generated the color chart having 100 color patches (10 × 10 color patches) using MATLAB as shown in Figure 3 [39], and took a picture of the color chart using a smartphone. The two-dimensional original image and its processed image are represented by ***O*** and ***P*** matrices, respectively, which are *i* × 3 matrices where ***i*** is the number of patches and 3 comes from the number of color channels containing R, G, B (red, green, blue) color channels (see Equation (1) below). Here, each patch consists of *m* rows (height) × *n* columns (width) pixels as shown in Figure 3.

(1) $$O=\left[\begin{array}{ccc} O_{1R} & O_{1G} & O_{1B}\\ \begin{array}{c} O_{2R}\\ \vdots \end{array} & \begin{array}{c} O_{2G}\\ \vdots \end{array} & \begin{array}{c} O_{2B}\\ \vdots \end{array}\\ O_{iR} & O_{iG} & O_{iB} \end{array}\right],\;P=\left[\begin{array}{ccc} P_{1R} & P_{1G} & P_{1B}\\ \begin{array}{c} P_{2R}\\ \vdots \end{array} & \begin{array}{c} P_{2G}\\ \vdots \end{array} & \begin{array}{c} P_{2B}\\ \vdots \end{array}\\ P_{iR} & P_{iG} & P_{iB} \end{array}\right].$$

Here, the individual terms in the *i* × 3 image matrices ***O*** and ***P*** are denoted by $O_{xy}$ and $P_{xy}$, respectively, where *x* varies in the range from 1 to *i* and *y* may be R, G, or B. $O_{xR}$, $O_{xG}$, and $O_{xB}\;$are the red, green, and blue intensities of the $x^{th}$ original image patches, and $P_{xR}$, $P_{xG}$, and $P_{xB}\;$are the red, green, and blue intensities of the processed image patches, respectively. 

**Figure 4:**
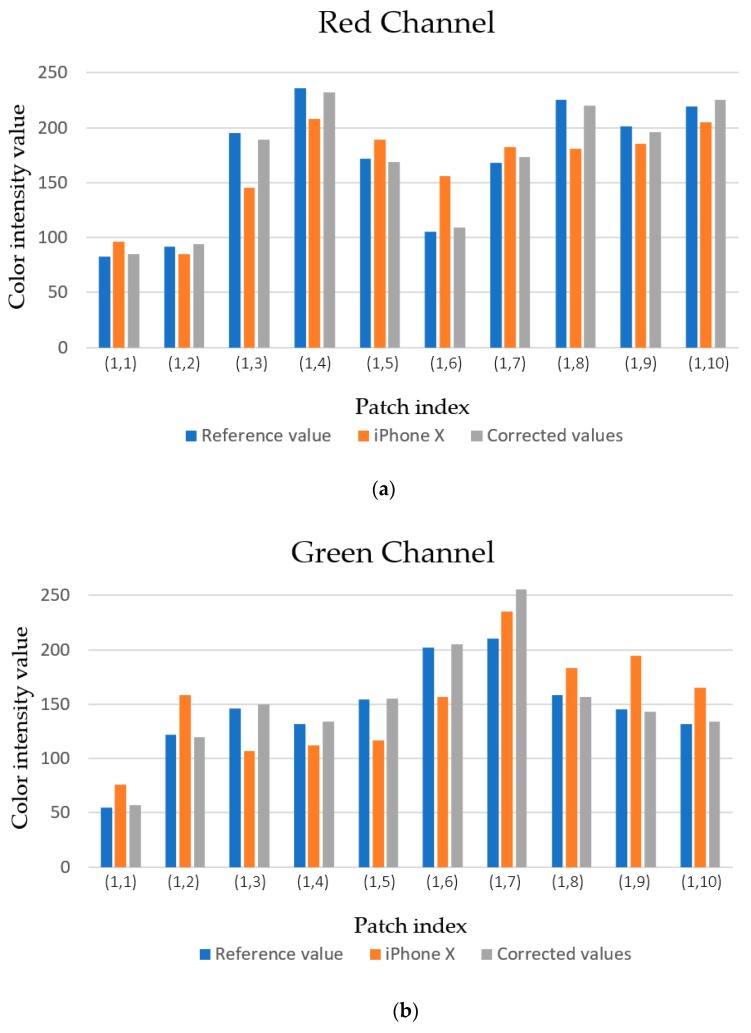

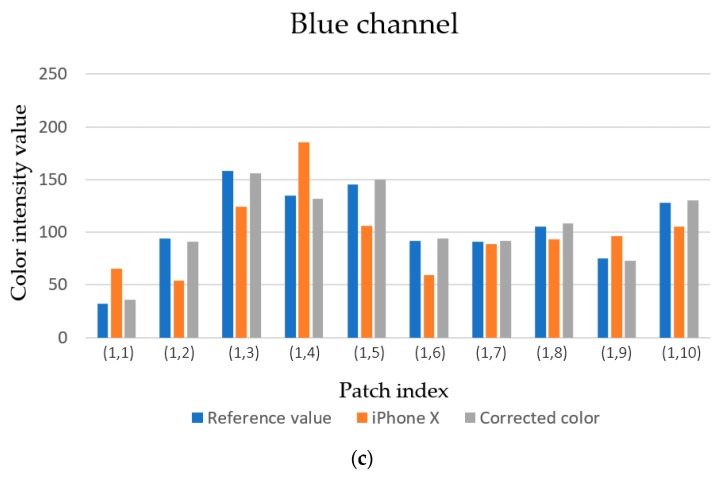
Comparison of R, G, and B values among reference, iPhone X, and color-corrected patches. Here, 10 color chart patches are chosen from 100 patches. (**a**) Red channel values, (**b**) green channel values, and (**c**) blue channel values.

**Figure 5:**
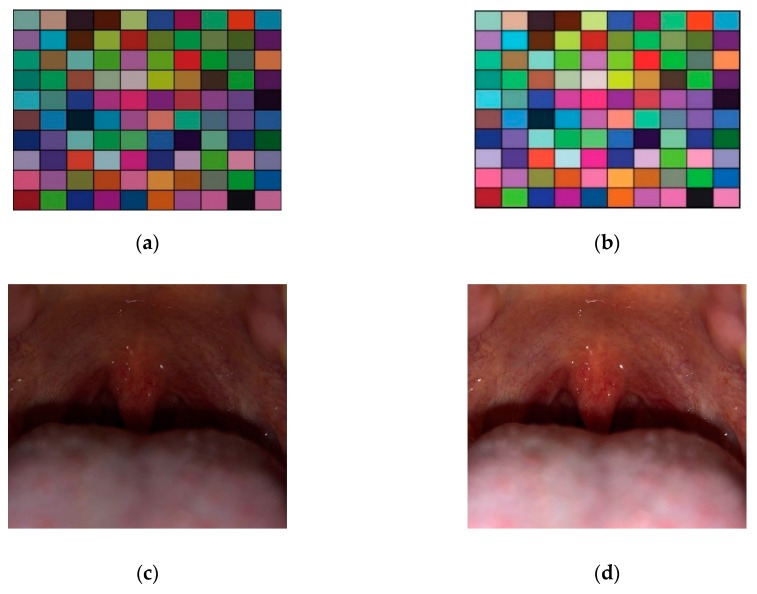
Output examples obtained by the color correction step of our proposed method. (**a**) Original image of the color chart, (b) corrected image of the color chart after applying our color corrected method, (**c**) original throat image which is presented by the color space in (a), and (**d**) color corrected throat image.

Denoting by ***A*** the color correction matrix, ***O*** can be expressed by ***A*** and ***P*** as follows:

(2) $$O=\left[\begin{array}{ccc} O_{1R} & O_{1G} & O_{1B}\\ \begin{array}{c} O_{2R}\\ \vdots \end{array} & \begin{array}{c} O_{2G}\\ \vdots \end{array} & \begin{array}{c} O_{2B}\\ \vdots \end{array}\\ O_{iR} & O_{iG} & O_{iB} \end{array}\right]=\left[\begin{array}{cc} 1 & P \end{array}\right]A=\left[\begin{array}{cccc} 1 & P_{1R} & P_{1G} & P_{1B}\\ 1 & P_{2R} & P_{2G} & P_{2B}\\ \vdots & \vdots & \vdots & \vdots \\ 1 & P_{iR} & P_{iG} & P_{iB} \end{array}\right]\left[\begin{array}{ccc} A11 & A12 & A13\\ A21 & A22 & A23\\ \begin{array}{c} A31\\ A41 \end{array} & \begin{array}{c} A32\\ A42 \end{array} & \begin{array}{c} A33\\ A43 \end{array} \end{array}\right],$$

where **1** denotes the column vector consisting of *i* rows of 1s. By adding column **1** to ***P***, a DC offset is added. Due to the appended **1** column vector with the matrix ***P****,* $A_{11}$*,* $A_{12}$*,* and $A_{13}$ were added in ***A*** to determine the optimal color offset. The product of $x^{th}$ row of the processed image (1, $P_{xR},\;P_{xG},\;P_{xB})$ and the first column of matrix ***A*** ($A_{11},\;A_{21},A_{31},\;A_{41})$ becomes $O_{xR}$. Similarly, $O_{xB}$ (or $O_{xG}$) is can be expressed by the product of $x^{th}$ row of matrix ***P*** and the second (or the third column) of matrix ***A*.** Color correction matrix ***A*** is calculated using the following equation [38]:

(3) $$A={\left({\left[\begin{array}{cc} 1 & P \end{array}\right]}^{T}\left[\begin{array}{cc} 1 & P \end{array}\right]\right)}^{-1}{\left[\begin{array}{cc} 1 & P \end{array}\right]}^{T}O,$$

where ${\left[\cdot \right]}^{T}$ stands for the transpose of a matrix. The color correction of 10 patches are presented in Figure 4. In Figure 4, (·,·) below each tick label on the *x*-axis indicates the location of the patch. e.g., (1,2) indicates the patch located at the 1^st^ row and 2^nd^ column. The corrected color values (gray bar) from the iPhone X color value (orange bar) became similar to the reference values (blue bar) after the color correction step as shown in Figure 4. The output examples obtained by this color correction step of our proposed method are shown in Figure 5. 

#### 2.2.2. Image Segmentation

**Figure 6:**
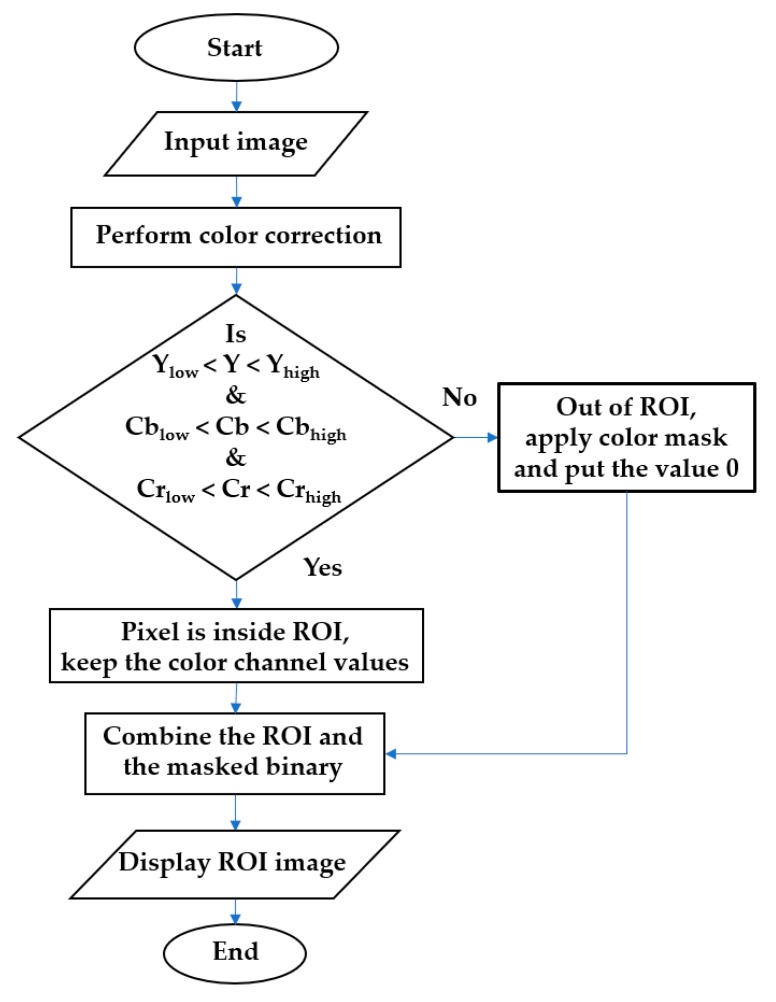
Flowchart of the image segmentation step of our proposed method. The image segmentation step extracts the region of interest (ROI) from the original image.

**Figure 7:**
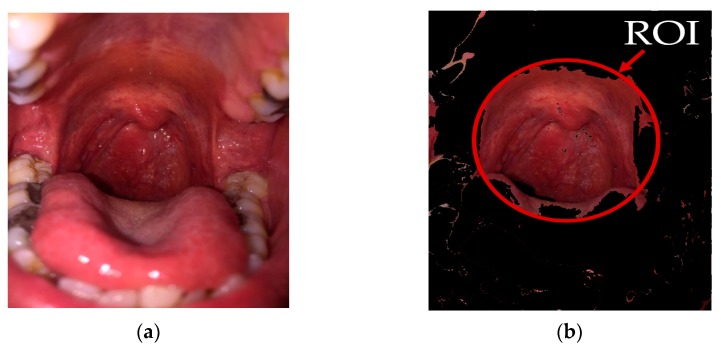
The example output obtained by the image segmentation step of our proposed method. (**a**) Original image, and (**b**) the ROI extracted from the original image in (**a**).

In the throat images acquired by the smartphone, there were five regions: (1) tongue, (2) palate, (3) lip, (4) teeth, and (5) throat tissue of the inner mouth. The image segmentation step is aimed at acquiring only the throat tissue region, which is the ROI in this paper, among the five regions in the input image. Since the color of the ROI was different from the other regions, we used the color intensity thresholding algorithm to find the ROI [40]. Specifically, we converted a raw RGB image obtained from the smartphone into a YCbCr image. Next, we extracted Y, Cb, and Cr channels, and finally, applied threshold values into each channel to find the ROI. Figure 6 shows the flowchart of the proposed color intensity thresholding algorithm to extract the ROI. The color intensity values of Y, Cb, and Cr channels were extracted from the color corrected image obtained in Section 2.2.1. We set the color intensity threshold values of Y, Cb, and Cr channels considering the ranges of color intensity values of ROI’s Y, Cb, and Cr channels. Specifically, the minimum and the maximum values of ROI’s Y, Cb, and Cr color intensity values were extracted to determine the corresponding threshold values of each channel. Denoting by *Y*_low_, Cb_low_, and Cr_low_ low threshold values of ROI’s Y, Cb, and Cr channels and denoting by *Y*_high_, Cb_high_, and Cr_high_ high threshold ones, the pixels which satisfied the following conditions are considered to constitute the ROI. Otherwise, the other pixels were considered to constitute non-ROI region as shown in Figure 6.

(4) $$R_{a}\left(r,c\right)\;=\{\begin{array}{l} R_{b}\left(r,c\right)\;\;\;\mathrm{if}\;\;\;Y_{\mathrm{low}}<Y<Y_{\mathrm{high}},\;Cb_{\mathrm{low}}<Cb<Cb_{\mathrm{high}},\;Cr_{\mathrm{low}}<Cr<Cr_{\mathrm{high}}\\ 0\;\;\;\;\;\;\;\;\;\;\;\;\;\;\;\mathrm{otherwise}, \end{array},$$

where $R_{b}\left(r,c\right)$ and $R_{a}\left(r,c\right)$ are color intensity values at the pixel location at *r*^th^ row and *c*^th^ column before and after the image segmentation step, respectively. Figure 7b shows an example of the ROI selection obtained by the image segmentation step of our proposed method on the throat image of Figure 7a.

### 2.3. Feature Extraction 

**Figure 8:**
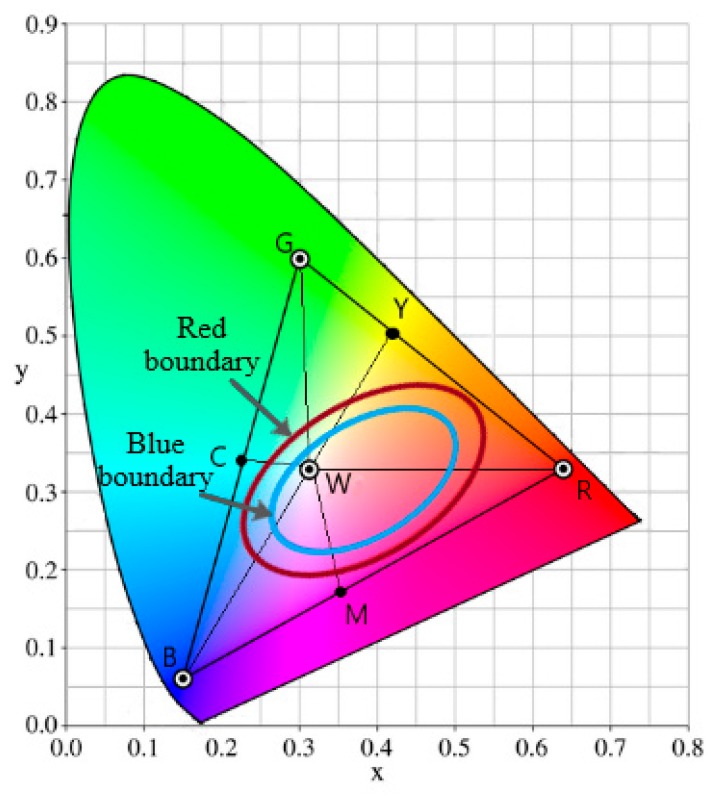
Throat color gamut. Over 98% of the throat color is in the blue boundary. The R, G, B, Y, C, M, and W stands for pure red, green, blue, yellow, cyan, magenta, and white colors, respectively [42].

Strep throat symptoms include red spots on the roof of the mouth, red and swollen tonsils, and white and yellow dots on the tonsils and the back of the mouth. These symptoms are the indications of bacterial inflammation. Hence, our proposed method extracts these features to detect strep throat symptoms [12,13,41]. Our method was designed and implemented to only distinguish strep throats from healthy ones. We first introduced throat color gamut and throat color features. We then used these color features to distinguish the strep throat images from healthy ones. All possible colors representing the throat surface are mainly distributed in the red and blue boundaries of Figure 8 [42]. The blue one provides the tighter boundary which covers almost 98% of the points of the throat surface. The colors that exist inside the blue boundary are the colors in the YCbCr range of the ROI mentioned in Section 2.2.

### 2.4. Classification

**Figure 9:**
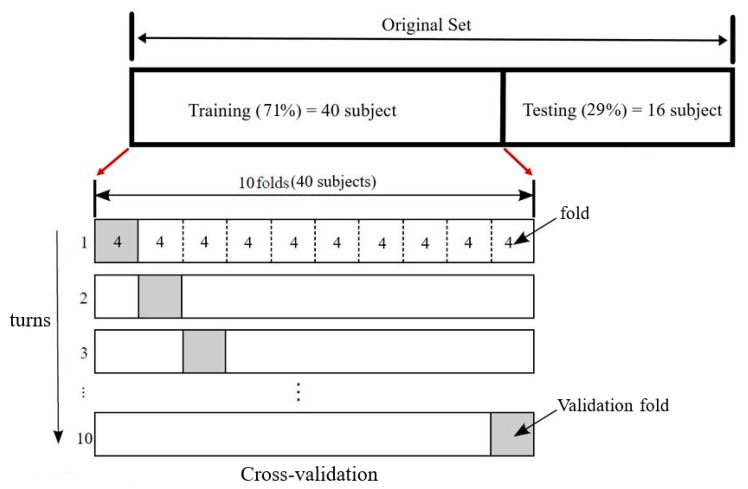
10-fold cross-validation technique used in our proposed method. The original data set was split into training (71%) and testing (29%). We applied 10-fold cross-validation to the training data set by dividing it into 10 folds (each fold contained four subjects). Specifically, 9 folds were used for training and the remaining 1-fold was used for validation. The cross-validation step was repeated 10 turns, rotating the training and validation folds.

We applied the *k*-NN classifier to distinguish strep throats from healthy throats since it is widely used in various fields such as medical imaging for brain tissue segmentation, MRI (magnetic resonance imaging) image classification, skin and breast cancer cell classification, and tongue image classifications due to its accuracy, fastness, and simplicity [43,44,45,46]. The *k*-NN classifier has also been shown to be compatible with running on smartphones [47]. We divided 56 data sets into 40 training and 16 test sets. This division was done in a random way to avoid bias [48,49]. Forty training sets consisted of 20 healthy subject images and 20 strep throat images. For the validation step, we adopted a *k*-fold cross-validation technique to prevent over-fitting. Specifically, we adopted 10-fold cross-validation which divided the data set into ten subsets and iteratively trained the algorithm on 9 folds while using the remaining fold as the validation set. Hence, the algorithm was trained on 9 folds (36 subjects) and the remaining set (four subjects) was left out for validation. This step was repeated for 10 turns (iterations) as shown in Figure 9. As a result of the 10-fold validation, we found the optimal parameter value *k* of the *k*-NN classification algorithm. As mentioned, 16 subjects (eight from healthy class and eight from diseased class) were left out for the test data set. We applied the decision boundary determined by this optimal parameter to the 16-test data set shown in Figure 9. 

## 3. Results

**Figure 10:**
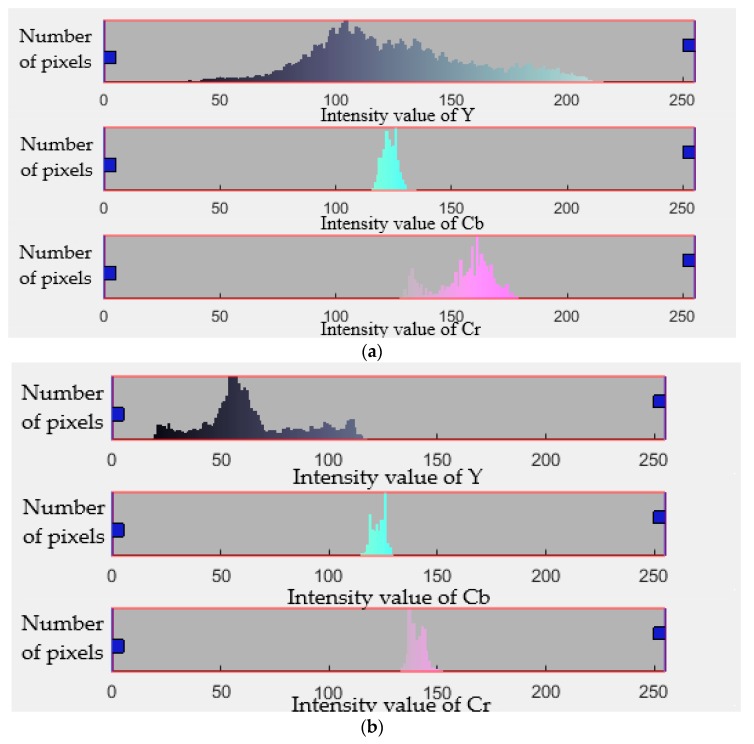
Histograms of Y, Cb and Cr color features of the acquired images. (**a**) Healthy throat color component histograms, and (**b**) diseased throat color component histograms. Here, the x-axis shows the intensity value of each color channel while the y-axis shows the number of pixels.

**Figure 11:**
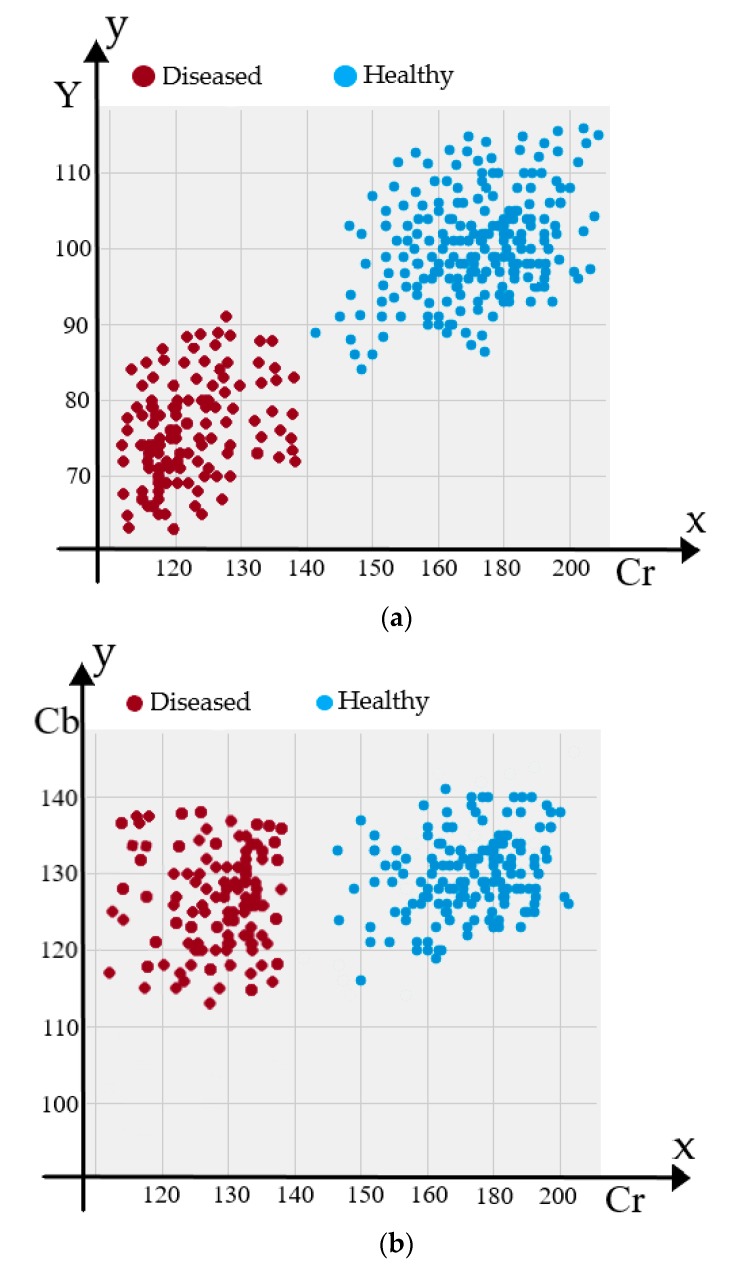
Color distribution of different color channels in healthy and diseased throats: (**a**) Y and Cr color intensity distribution of healthy and strep throats, and (**b**) Cb and Cr intensity distribution of healthy and strep throats.

We evaluated the performance of our proposed smartphone-based strep throat detection method by calculating accuracy, sensitivity, and specificity when the detection algorithm was applied to throat images of 56 subjects. We derived the color gamut of the throat area where three color features Y, Cb and Cr were extracted. The histograms of Y, Cb and Cr components values of healthy and strep throats are shown in Figure 10a,b, respectively. The mean values of the color components (channels) for the healthy throat and strep throats were derived and represented in Table 1. Figure 11 shows the color distribution of the Y, Cb, and Cr color channels. The distribution of Y-Cb color channels is shown in Figure 11a while the color distribution of the Cb-Cr channels is shown in Figure 11b. As shown in Table 1 and Figure 11, Cb values are similar between healthy and strep throats while Y and Cr values were noticeably different between healthy and strep throats.

**Figure 12:**
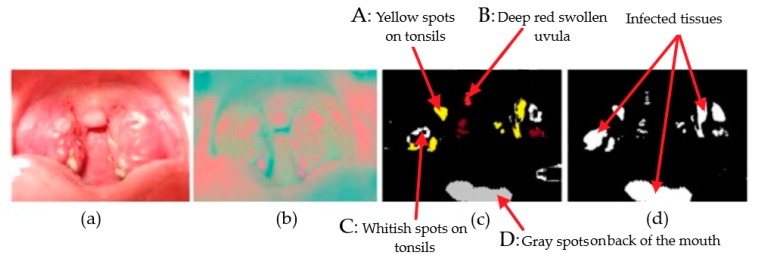
Strep detection procedure based on YCbCr components of a throat image: (**a**) Original RGB image, (**b**) YCbCr image converted from the RGB image in (**a**), (**c**) infected tissue detected in (**b**), and (**d**) infected tissue marked in white color.

Figure 12 shows an example of the strep detection procedure. The acquired RGB image is shown in Figure 12a. Figure 12b shows the YCbCr image converted from RGB image in Figure 12a. Figure 12c,d show the infected tissue detected in Figure 12b and in white colors, respectively. The colors that we were seeking as symptoms of the strep throat have been in Figure 12. The strep tissue are indicated by A, B, C, and D symbols in Figure 12 and the color intensity values of the infected tissue have been represented in Table 2. A paired-*t* test was performed to compare the average Y, Cb, and Cr values of healthy and diseased throats. The significant difference test was performed on the parameter value $YCbCr_{avg}$=$\;\frac{Y+Cb+Cr}{3}$ which has been proven to be effective in distinguishing healthy and diseased tissue with bacterial infection [17,32,34]. The paired-*t* test indicated that the $YCbCr_{avg}$=$\;\frac{Y+Cb+Cr}{3}$ from the healthy throat (mean = 146.3, STD = 6.8) was significantly higher than diseased ones (mean = 124.4, STD = 5.1) with p=0.04. Specifically, the values of mean difference and standard deviation of difference were 21.9 and 5.6, respectively.

**Figure 13:**
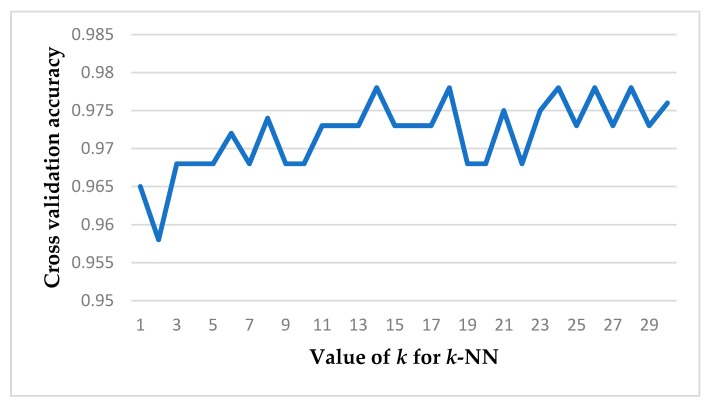
Cross-validation accuracy for varying *k* values of the *k*-NN classifier from 1 to 30. As *k* value increases, the accuracy value of cross validation increases while the processing takes more time. The optimal *k* value was achieved at *k* = 13 in terms of cross validation accuracy and processing time.

We divided the data (56 subjects) into a training and validation set (40 subjects), and a test set (16 subjects). Here, for the training and validation set (40 subjects), 20 healthy and 20 strep subjects were randomly chosen from the total 56 subject data to avoid biasing [48]. As a result of 10-fold validation, we found the optimal *k* value for the *k*-NN classifier is 13 since it gives the highest accuracy as shown in Figure 13. We applied the decision boundary determined by this optimal *k* value (*k* = 13) to the test data set (16 subjects). 

As performance metrics, we considered accuracy, sensitivity, and specificity which were calculated using true positive (TP), true negative (TN), false positive (FP), and false negative (FN) values as follows:

(5) $$Sensitivity=\;\frac{TP}{TP+FN}\times 100\%,\;$$

(6) $$Specificity=\;\frac{TN}{TN+FN}\times 100\%,\;$$

(7) $$Accuracy=\frac{TP+TN}{TP+TN+FP+FN}\times 100\%,\;$$

where *TP*, *FP*, *TN*, and *FN* were counted in terms of the number of images. Since the scope of this paper was confined to binary classification between strep and healthy throats as mentioned in Section 2, *TP*, *FP*, *TN*, and *FN* were calculated considering this binary classification. That is, *TP* is the number of images which were correctly determined to be strep given that they are strep, and *FP* is the number of images which were incorrectly determined to be strep given that they are healthy. On the other hand, *TN* is the number of images which were correctly determined to be healthy given that they are healthy, and *FN* is the number of images which were incorrectly determined to be healthy given that they are strep.

The average accuracy of the 10-fold cross-validation was calculated by averaging the accuracy values of all turns (iterations) of the cross-validation. Table 3 shows the average accuracy, sensitivity, and specificity values of the proposed algorithm. The average and standard deviation value of the cross-validation accuracy was 97.8% ± 0.014% as shown in Table 3. We applied the decision boundary obtained from this 10-fold cross-validation into the test data set (8 healthy and 8 strep throat images). As a result, we obtained 93.75% accuracy, 87.5% sensitivity, and 88% specificity, for the test dataset as shown in Table 3. 

**Figure 14:**
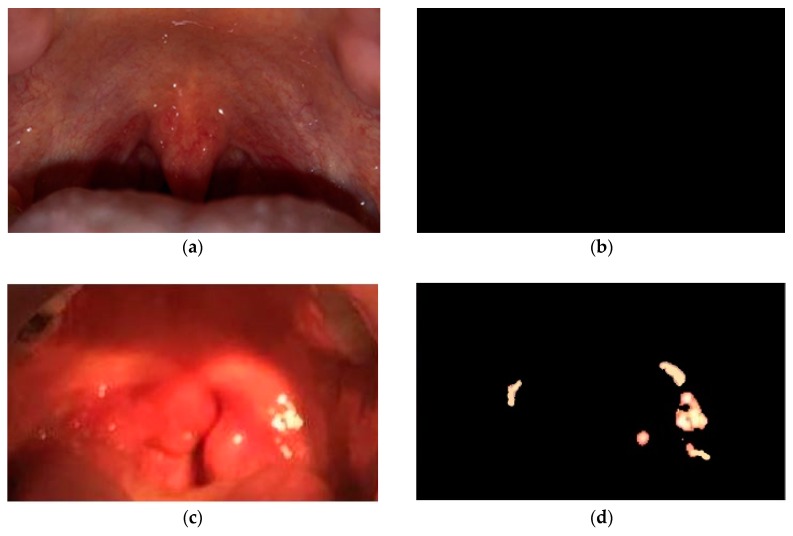
Detection of strep throat tissue using the proposed method. (**a**) Original image of a healthy throat, (**b**) the throat in (a) is diagnosed to be healthy (no infected tissue is detected in the image), (**c**) original image of a diseased throat, and (**d**) the throat in (c) is diagnosed to be infected (infected tissue is marked in bright color).

Figure 14 shows example outputs of our proposed method on one healthy throat and one strep throat. Figure 14a is the original image from the healthy throat and Figure 14b is the result of our method on the healthy throat. Figure 14c is the original image from strep throat and Figure 14d is the result of our method on the strep throat. Infected tissue are detected in the strep throat as shown in Figure 14d while those are not detected in the healthy throat as shown in Figure 14b.

## 4. Conclusion and Discussion

In this paper, we have investigated the plausibility of using a smartphone to detect strep throat by evaluating our developed smartphone-based strep throat detection method on subjects’ throat images taken by a smartphone camera. We recruited 56 subjects consisting of 28 strep and 28 healthy subjects, acquired subjects’ throat images using an iPhone X, and tested our method on them. The aim of the proposed method was to find symptoms (color features) that indicate the signs of streptococcal pharyngitis in the throat. To improve the performance of our proposed method, we designed and manufactured an add-on gadget to control the lighting conditions and avoid ambient light and reflection. We proposed the use of color intensity thresholding techniques to segment throat tissue from a throat image. In this paper, a novel least square color correction method and YCbCr color space that is luminance-independent (by extracting Y channel) has been proposed. The color intensity thresholding technique has been applied and evaluated in detecting tongue color as well [50]. However, they had different approaches in evaluating their color intensity-based techniques. For example, a support vector machine (SVM) was adopted as a classifier to distinguish diseased subjects from healthy ones in Refs. [17,31,32,33,34,44]. We adopted a *k*-NN classifier as in Refs. [31,44] and evaluated the performance using *k*-fold validation approach as in Refs. [17,32,33,34]. The experimental results have shown that the proposed color intensity thresholding system could segment throat image tissue in a throat image. We have simplified the categories of throat images into strep and healthy throats since the scope of this paper was not the multiclass classification of different degrees of strep (or *streptococcal pharyngitis*) but was confined to binary classification between strep and healthy throats. Cross-validation was performed to prevent overfitting. Here, 10-fold cross-validation was specifically adopted. After running 10-fold cross-validation on a range *k* from 1 to 30 for the *k*-NN classifier, the highest validation accuracy 97.8% was achieved at *k* = 13. The experimental results have shown that the proposed method detects strep throat with 97.8% average accuracy (validation score) for the 10-fold cross-validation training data set. Using the *k*-NN classifier, the proposed strep detection method can detect strep from the throat tissue with 93.75% accuracy, 87.5% sensitivity, and 88% specificity for the testing dataset. This method can be implemented using any smartphone, including iOS or Android phones with an appropriate add-on gadget using a retargetable application platform [51]. Extending this result into classifying different degrees of strep throat and differentiating bacterial from viral infections can be considered in future work.

## Figures and Tables

**Table 1:** Mean, standard deviation, and the range of the color intensity values of healthy and strep throats.

Color Channel	Y	Cb	Cr
Healthy (Mean ± STD)	133.5 ± 12	127 ± 5	168.5 ± 11
Diseased (Mean ± STD)	97 ± 5	137 ± 6	141 ± 8
Healthy (range)	122–145	112–142	155–185
Diseased (range)	92–103	118–132	135–147

**Table 2:** Mean and standard deviation of $YCbCr_{avg}$ values in the A, B, C, and D regions from all healthy and diseased throats.

Strep Throat Symptoms	$\mathrm{Healthy}\;YCbCr_{avg}$ (Mean ± STD)	$\mathrm{Disease}\;YCbCr_{avg}$ (Mean ± STD)
A in Figure 12	154 ± 6.8	141 ± 4.3
B in Figure 12	165 ± 7.6	143 ± 5.1
C in Figure 12	136.2 ± 4.4	152.6 ± 6.7
D in Figure 12	151.2 ± 6.6	134.6 ± 5.4

**Table 3:** Average accuracy, sensitivity, and specificity values of the proposed method.

Cross Validation Accuracy (Mean± STD)	Average Test Accuracy	Average Test Sensitivity	Average Test Specificity
0.978 ± 0.014	0.9375	0.875	0.88
